# Traditional lifestyles, transition, and implications for healthy aging: An Example from the remote island of Pohnpei, Micronesia

**DOI:** 10.1371/journal.pone.0213567

**Published:** 2019-03-12

**Authors:** Michael J. Balick, Roberta A. Lee, Jillian M. De Gezelle, Robert Wolkow, Guy Cohen, Francisca Sohl, Bill Raynor, Clay Trauernicht

**Affiliations:** 1 Institute of Economic Botany, The New York Botanical Garden, Bronx, New York, United States of America; 2 Southern Arizona VA Health Care System, Tucson, Arizona, United States of America; 3 Department of Plant and Microbial Biology, North Carolina State University, Raleigh, North Carolina, United States of America; 4 American Academy of Family Physicians, Leawood, Kansas, United States of America; 5 Department of Statistics, Columbia University, New York, New York, United States of America; 6 Conservation Society of Pohnpei, Kolonia, Pohnpei, Federated States of Micronesia; 7 Formerly, Indo-Pacific Division of The Nature Conservancy, Kolonia, Pohnpei, Federated States of Micronesia; 8 Department of Natural Resources and Environmental Management, University of Hawaii at Manoa, Honolulu, Hawaii, United States of America; Scripps Florida, UNITED STATES

## Abstract

Lifestyle-related, non-communicable diseases, such as diabetes, hypertension, and obesity have become critical concerns in the Pacific islands of Micronesia. We investigated the relationship between the diminution of traditional lifestyle practices and the decline in the health of the population in the State of Pohnpei, Federated States of Micronesia. To assess this, our interdisciplinary team developed two scales, one to rank individuals on how traditional their lifestyles were and one to rank individuals on the healthiness of their lifestyles. Participants’ locations were categorized as living on a remote atoll, living on the main island, or as a transitional population. Pohnpeians living in transitional communities (e.g. recently moved from a remote atoll to the main island, or the reverse) ranked lowest on both the tradition and health scales, rather than ranking intermediate between the remote and main island groups as we had hypothesized. As predicted, individuals residing on the remote atolls were living the most traditional lifestyles and also had the healthiest lifestyles, based on our rating system. The higher an individual scored on the tradition scale, e.g. the more traditional life they lived, the higher they scored on the health scale, suggesting the importance of traditional lifestyle practices for maintaining health. These findings have significant implications for promoting health and longevity of Micronesians and other Pacific Island peoples. We suggest the process of transition be recognized as a significant lifestyle and health risk and be given the attention we give to other risk factors that negatively influence our health. Based on our findings, we discuss and recommend the revitalization of particular traditional lifestyle practices, which may advance healthy aging among Pohnpeians.

## Introduction

As the world’s population develops, embracing modernization, technological advancement, increased communication, contact, and international trade, indigenous peoples living traditional lifestyles, once unaffected by such processes, now must confront powerful global change. The health effects of transitioning from traditional lifestyles toward westernized or modernized lifestyles are only recently beginning to be identified [1–3]. The implications on aging and longevity are even less well understood. Studies comparing lifestyle among traditional, transitional, and modern populations who share genetic lineage can elucidate the health effects of transition and modernization on populations as they depart from traditional practices, lifestyles, and values.

The Federated States of Micronesia (FSM), located in the Western Pacific Ocean, is a nation in transition—from traditional lifestyles to those heavily influenced by imported practices and products. Pohnpei, the largest of the four states comprising the FSM (along with the other three states of Chuuk, Kosrae and Yap), and the location of our study, is today experiencing the adverse health effects of such lifestyle changes. The Annual Health Services Profile [4] distributed by the Pohnpei State Department of Health Services reported that diabetes and diabetes-related problems—which include hypertension, cardiac diseases and cancer—remain major health concerns in Pohnpei. Type II diabetes mellitus is the leading cause of hospitalization, followed by hypertension. Diabetes mellitus and diabetes-related complications were the leading causes of deaths in Pohnpei. The report noted that while diabetes is "largely a life-style related and a controllable and preventable disease, very minimal resources [are] expended to primary prevention” in Pohnpei. The Pohnpei State Department of Health Services considers macro and micronutrient malnutrition (including Vitamin A deficiency, anemia and iron deficiency, and marasmus) also to be persistent public health challenges and priorities. The report stated that “high rates in Pohnpei reflect our nutritional profile and eating/feeding habits. Sadly, [the] majority of these conditions are preventable by eating locally available foods.”

The World Health Organization (WHO) has developed a system for classifying individuals in risk categories for noncommunicable diseases (NCDs), utilizing five common and critical NCD risk factors. The risk factors are: 1) overweight, 2) daily smoker, 3) low levels of physical activity, 4) fewer than five servings of fruits and vegetables per day, and 5) elevated blood pressure. Using this classification system, only 1% of their study population of 1,638 Pohnpeians surveyed in 2002 were found to be at low risk for NCDs (0 risk factors of the 5), while 42.3% were found to be at moderate risk (1–2 risk factors), and 56.7% were found to be at high risk (3–5 risk factors) [5]. Among the youngest group surveyed, 25–44 year olds, 52.6% were already at a high risk for NCDs, mirroring the alarming transition of younger populations in the U.S. who are becoming markedly obese.

By 2050, the Western Pacific region will have the second oldest population of all regions recognized by the World Health Organization [6]. Populations of the elderly are growing faster in the developing world than in much of the developed world, and often these individuals have limited economic resources. Due to changes in lifestyle, environment, and lack of access to health care resources, many diseases facing the older population are now chronic, non-communicable diseases (e.g. hypertension, heart disease and diabetes), while much of the medical attention given to developing countries in the past has been devoted to the control of communicable diseases such as tuberculosis or malaria [6]. With many younger people migrating to cities or abroad for work or study, smaller families with lower birthrates, and more women in the workforce, elderly family members may remain in the rural areas with a much smaller support network of kin to care for them [6]. Promoting the benefits of maintaining traditional lifestyle practices for Micronesians and those in other parts of the Western Pacific is critical for ensuring healthy aging amidst cultural and environmental degradation.

Our study investigated diet, lifestyle, traditional knowledge and several ethnobotanical and ethnomedical variables, and their relationship to health and wellbeing. The research design was based on the belief that the study of isolated island populations in transition from traditional to modern or westernized lifestyles, as found in Micronesia, provides an ideal and unique opportunity for investigating the effects of lifestyles on health outcomes. The study was driven by the hypothesis that a traditional lifestyle, which incorporates healthy diet, community relationships, and regular physical exercise, promotes healthy aging and longevity in the Micronesian population. We chose to evaluate lifestyle health using a questionnaire based on: 1) participant self-evaluation of health, and 2) questions indicating healthy and unhealthy practices. The survey was designed by our multidisciplinary team of research scientists, medical doctors, educators, and local community members. This study was part of the “Biodiversity and Human Health in Micronesia” project investigating traditional uses of plants for many cultural purposes, including food and medicine, with the goal of conserving this knowledge, along with the biological diversity that it depends upon [7].

## Materials and methods

### Research ethics

This study was carried out in partnership with local governmental organizations and traditional leaders. When seeking permission to carry out this study on Pohnpei, FSM, we requested and received permission and guidance from directors of the following institutions: Micronesia Ministry of Health, the Pohnpei State Department of Health Services, the Pohnpei State Government, and the Pohnpei State Hospital. Because of local protocols, permission was requested from and granted by the *Mwoalen Wahu Ileilehn Pohnpei* (Pohnpei Council of Traditional Leaders), the body that reviews and grants permission for culturally-related activities carried out on Pohnpei. In the absence of a local Institutional Review Board at the time of this study, the local governmental institutions carried out this function, carefully reviewing questionnaires, making suggestions and providing parameters for this survey. One of the parameters was an informed consent statement in Pohnpeian. Each participant gave verbal and written consent to participate in the study.

### Study sites and classification

**Fig 1:**
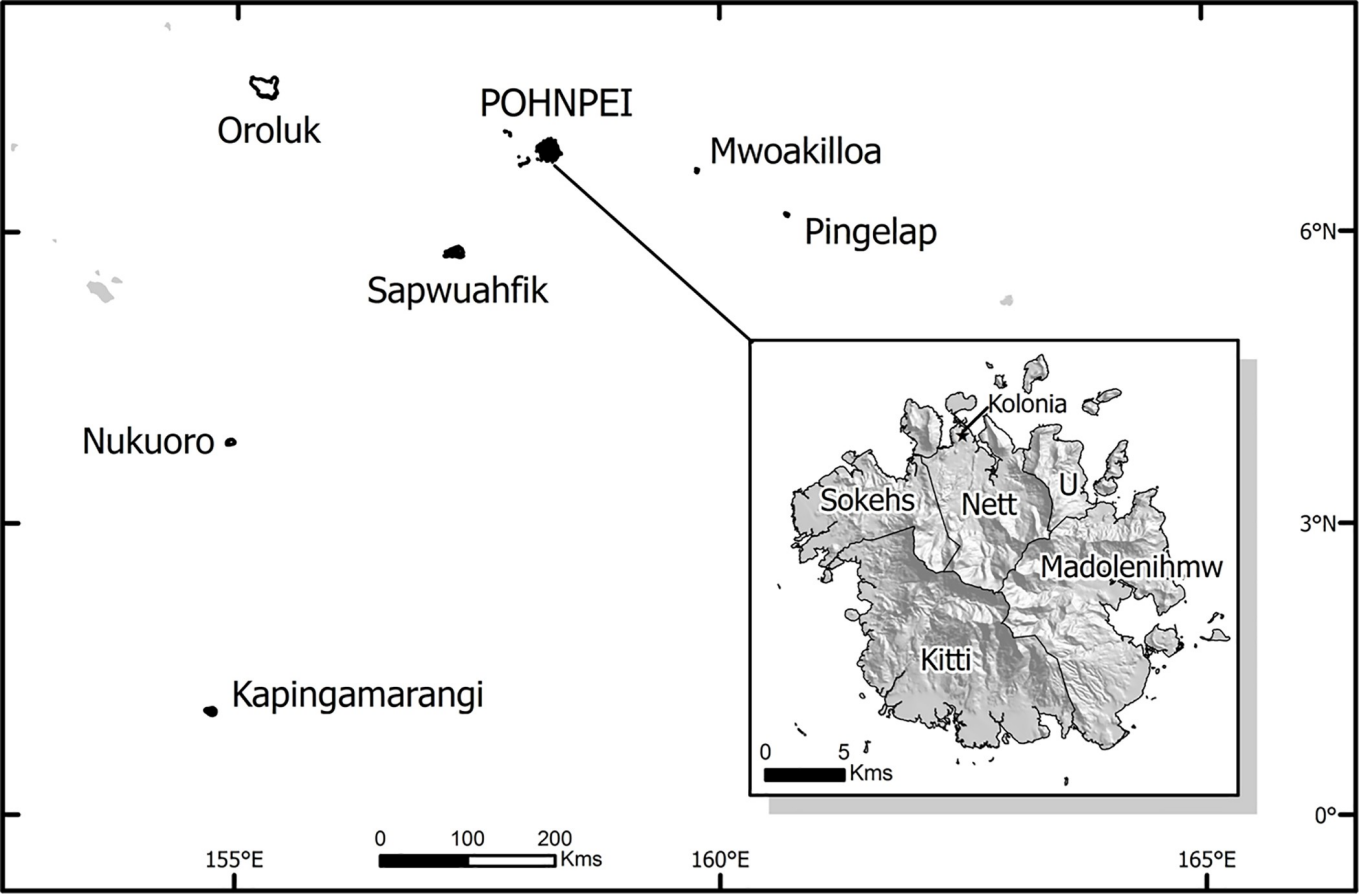
Map of Pohnpei including Pohnpeian atolls. This map shows the island state of Pohnpei and its surrounding atolls. The inset image shows the five municipalities or kingdoms of Pohnpei and the capital, Kolonia. Map courtesy of Hannah Stevens, Geographic Information Systems Laboratory, The New York Botanical Garden.

Study participants’ locations were classified and ranked as 1) remote, 2) transitional, or 3) main island communities (Table 1). The main island of Pohnpei is divided into five municipalities or kingdoms: Sokehs, Nett, U, Madolenihmw, and Kitti (Fig 1). The capital of the State of Pohnpei is Kolonia, located in Nett on the northern end of the island of Pohnpei. Those living in one of the five municipalities in Pohnpei and those in the capital of Kolonia were considered to be living in main island communities.

**Table 1:** Municipality classification for study participants.

Municipality	Municipality Grouping	Sample
Mwoakilloa	Remote	41
Nukuoro	Remote	38
**TOTAL**		**79**
Kapingamarangi to Main Island	Transitional (Remote-Main Island)	1
Mwoakilloa to Main Island	Transitional (Remote-Main Island)	19
Nukuoro to Main Island	Transitional (Remote-Main Island)	3
Oroluk to Main Island	Transitional (Remote-Main Island)	1
Pingelap to Main Island	Transitional (Remote-Main Island)	3
Sapwuahfik to Main Island	Transitional (Remote-Main Island)	7
Sokehs to Mwoakilloa	Transitional (Main Island-Remote)	23
**TOTAL**		**57**
Kolonia (capital)	Main Island	32
Kitti	Main Island	38
Madolenihmw	Main Island	43
Nett	Main Island	52
Sokehs	Main Island	64
U	Main Island	42
**TOTAL**		**271**
**GRAND TOTAL**		**407**

The outer islands of Nukuoro and Mwoakilloa were considered to be remote communities (Fig 1). These are quiet, remote atolls with electricity available only by generator, and medical facilities consisting usually of a one-room medical dispensary equipped with a radio and approximately 30 different types of medications. Mwoakilloa can be reached by boat typically once every month or two, or by plane once a week. Those who had moved from Mwoakilloa, Nukuoro, or other atolls Kapingamarangi, Oroluk, Pingelap, and Sapwuahfik, to the main island, and those that moved from Sokehs, on the main island to the atoll of Mwoakilloa during their lifetime were considered to be transitional. Initial attempts were made to subset the transitional group between movement from main island to remote and movement from remote to main island. The sample size was not sufficient to permit this division in categorization.

### Interviews

Structured interviews were conducted in person with 407 inhabitants (1.3% of the population) of Pohnpei and the outer atolls by Pohnpeians in their native language, utilizing a survey with 121 questions. All interviews were preceded by reviewing and signing an informed consent document that explained the study. The detailed survey contained 21 questions pertaining to diet, 15 questions regarding transitional state, 14 questions regarding use of sakau (*Piper methysticum* G. Forst.), which is considered to be the most important plant to the traditional culture of Pohnpei, 13 questions pertaining to agriculture, 11 demographics questions, 10 questions regarding home and work life, 10 questions regarding traditional knowledge, 7 perceived health and healthcare-related questions, 6 questions eliciting opinions on tradition and transitioning, 5 questions about betel nut (*Areca catechu* L.) usage, 4 questions regarding sleep, 3 questions regarding physical activity, and 2 questions on community relationships.

This survey gathered data for a larger comprehensive study, though we utilized only the relevant components to answer our question regarding the role of traditional lifestyle in maintaining health. Forty-four 41FooFOquestions from the 121 question survey were selected by our multi-disciplinary team to create the scales for our analysis. The remainder of the survey questions were not relevant to the construction of the scales since they were either demographic in nature, used to elicit opinions in the form of short answers, or to elicit further details for the questions used in our scales. For instance, the question of whether or not an individual owns land was followed by questions as to whether they lived on the land, whether any of the land was in the mountain forest or any in the lowland area. Upon review of the surveys, participants were given a Traditional/Modern Score and a Healthy/Unhealthy Score based on the relevant 44 survey questions, according to the scales below.

Our two scales were developed independently through selection of relevant questions from the survey for inclusion in the analysis; the Healthy-Unhealthy Scale was developed by the medical professionals on our research team, and the Traditional-Modern Scale was developed by the Pohnpeian members of the research team. While there are a small number of similar or related questions found on both scales, such as drinking sakau with alcohol that was independently classified as both modern and unhealthy, and fishing which was independently classified as both traditional and healthy, these findings highlight the important relationship between lifestyle and health among the population.

### The Traditional-Modern scale

Lifestyle was assessed by classifying selected questions into two groups, representing what are considered “more traditional factors” versus “more modern factors.” In the Traditional-Modern Scale, for each response that was a “yes,” a 1 was assigned. The Traditional/Modern Score equals the sum of traditional factors minus the sum of modern factors. The more positive the number, the more traditional the individual is considered, based on this scale. A “yes” to each of the following questions was evaluated as a traditional “factor”:

1. Knows their Nahnmwarki’s name
2. Has a traditional title indicating societal rank
3. Has a traditional canoe
4. Family owns a canoe
5. Goes fishing
6. Follows the rules of the Nahnmwarki for fishing
7. Raises pigs for eating and to give as tribute to Chiefs
8. Has land
9. Grows crops on the land
10. House is made of traditional materials (local woods and grasses)
11. Pounds (prepares) sakau in the traditional way
12. Drinks freshly prepared sakau (made by pounding roots on the rock)
13. Uses traditional medicines
14. Built their own house, another house, or Nahs (feast house) with traditional materials

The following responses were each considered as a modern “factor”:

1. Working in a paying job to buy a car
2. Fishes but does not follow rules of the Nahnmwarki
3. Raises pigs for selling (vs. only for tribute)
4. Raises chickens for selling
5. Has a cement house
6. Works in a government job
7. Watches television
8. Goes to sakau bars, where sakau is not prepared traditionally
9. Drinks sakau followed by alcohol
10. Takes sakau pills (a form of concentrated extract)
11. Goes to a medical clinic
12. Chews betel nuts
13. Chews betel nuts with pepper leaf
14. Works on a computer
15. Knows how to type
16. Has a desk job
17. Rents videos

Some factors incorporated into the Traditional-Modern Scale require further explanation. The Nahnmwarki is the Paramount traditional Chief (also known as King) in Pohnpeian society. The other highest ranking Chief in the Kingdom is known as the Nahnken. A Pohnpeian who has a title has been acknowledged and given such title by a village or higher-ranking leader, which establishes their place in the hierarchy of traditional Pohnpeian societal structure. Fishing rules, determined by the Nahnmwarkis and community chiefs, state that certain fish are reserved only for the Nahnmwarki and should be brought to the Nahnmwarki if caught. These rules are not typically enforced anymore, though a small number of Pohnpeians still bring the biggest fish to the Nahnmwarkis. Few individuals still follow the fishing rules for line fishing, spear fishing, and net fishing.

Sakau is the Pohnpeian word for *Piper methysticum* G. Forst. (Piperaceae), known elsewhere in the Pacific and in Western herbal medicine as kava, a mildly intoxicating drink with anxiolytic properties, consumed traditionally on Pohnpei. If an individual “pounds sakau” they prepare the drink themselves (often from plants they grow in their fields) in the traditional manner by pounding the fresh roots on a flat stone. The traditional way to drink sakau is referred to as “on the rock,” meaning that it is traditionally prepared (by pounding the roots “on the rock”) and consumed according to customary ritual. According to the WHO survey conducted in 2002 [5] with 1,638 Pohnpeian participants, 79% of men and 59% of women had tried sakau. Our findings from a survey with 180 Pohnpeians in 2001 were similar. Of the total sample, 69% reported drinking sakau, and 83.6% of the males and 53.2% of the females drank sakau [8].

Sakau is also widely consumed in ways that do not follow traditional ritualistic protocol such as in sakau bars, which are open-air bars that serve sakau and sometimes alcohol. Sakau is also prepared for sale in one liter bottles (“market sakau”), sold in plastic coolers along the road and in parking lots of stores. Sakau is also occasionally ingested as a pill, which can be a capsule that contains pulverized *Piper methysticum* roots or a dietary supplement prepared from an extraction of the roots that concentrates the kavalactones, the active compounds in this plant. A project to produce sakau pills with a very high (up to 90%) kavalactone content once operated on Pohnpei, but is no longer active, although previously produced product continues to be sold in some places. Drinking sakau with alcohol refers to the practice of moderate to heavy alcohol (typically beer) consumption following a night of sakau drinking (known as “kapohpo”). All of these practices reflect a significant transition away from traditional use of sakau, where ritual took precedence over inebriation. Thus, a self-regulating cultural mechanism limited consumption.

Betel nut is the seed from the palm, *Areca catechu* L. (Arecaceae), which is used as a masticatory for its mild stimulating effects, but has been correlated with increases in oral cancer [9]. Betel nuts are chewed daily by approximately 40% of the men and 15% of the women on Pohnpei, and the highest proportions of betel nut chewers were in the youngest age group surveyed, 25–34 years, according to the 2002 WHO survey [5]. Some Pohnpeians chew betel nut wrapped with the leaf of the *Piper betle* L. (Piperaceae) plant. Powdered lime and often tobacco are also added to the chewing mixture. Chewing betel nut is a recent practice on Pohnpei.

### The Healthy-Unhealthy scale

Health was assessed by classifying selected questions into two distinct groups representing “more healthy factors” versus “more unhealthy factors.” In the Healthy-Unhealthy Scale, for each response that was a “yes,” a 1 was assigned. The Healthy/Unhealthy Score equals the sum of healthy factors minus the sum of unhealthy factors. The more positive the number, the healthier the individual is considered, based on this scale. A “yes” to each of the following questions was evaluated as a healthy “factor”:

1. Goes fishing often
2. Eats fresh fish
3. Grows plants, including sakau, vegetables, fruits
4. Does not watch television
5. Does not drink sakau with alcohol
6. Eats taro, breadfruit and greens grown in the garden
7. Does not eat white rice, pizza, candy, canned vegetables, Spam, or white bread
8. Considers themselves healthy
9. Does not chew betel nut with or without lime
10. Eats coconut
11. Exercises frequently

The following responses were each considered as an unhealthy “factor”:

1. Rarely fishes
2. Eats canned fish more than 5 times a week
3. Does not grow food on their land
4. Watches TV more than 2 hours a week
5. Drinks sakau with alcohol
6. Does not eat taro or breadfruit or coconut
7. Eats white rice, candy, pizza, or white bread
8. Does not have a taro patch
9. Considers themselves unhealthy
10. Chews betel nut with or without lime
11. Does not have friends
12. Does not exercise

### Statistical methodology

The demographic data and subject characteristics were summarized based on the three municipality classifications. The mean score for the defined scales was summarized for each of the three municipality groupings. The Pearson Correlation coefficient was calculated between the Traditional-Modern Scales and the Healthy-Unhealthy Scales. Point estimates were presented with 95% confidence intervals. For percentages, the normal approximation to the binomial estimate was used. The survey data were entered into an EXCEL (Microsoft) database. These data were exported in SAS version 8 (SAS Institute). All data were processed using SAS.

## Results

**Fig 2:**
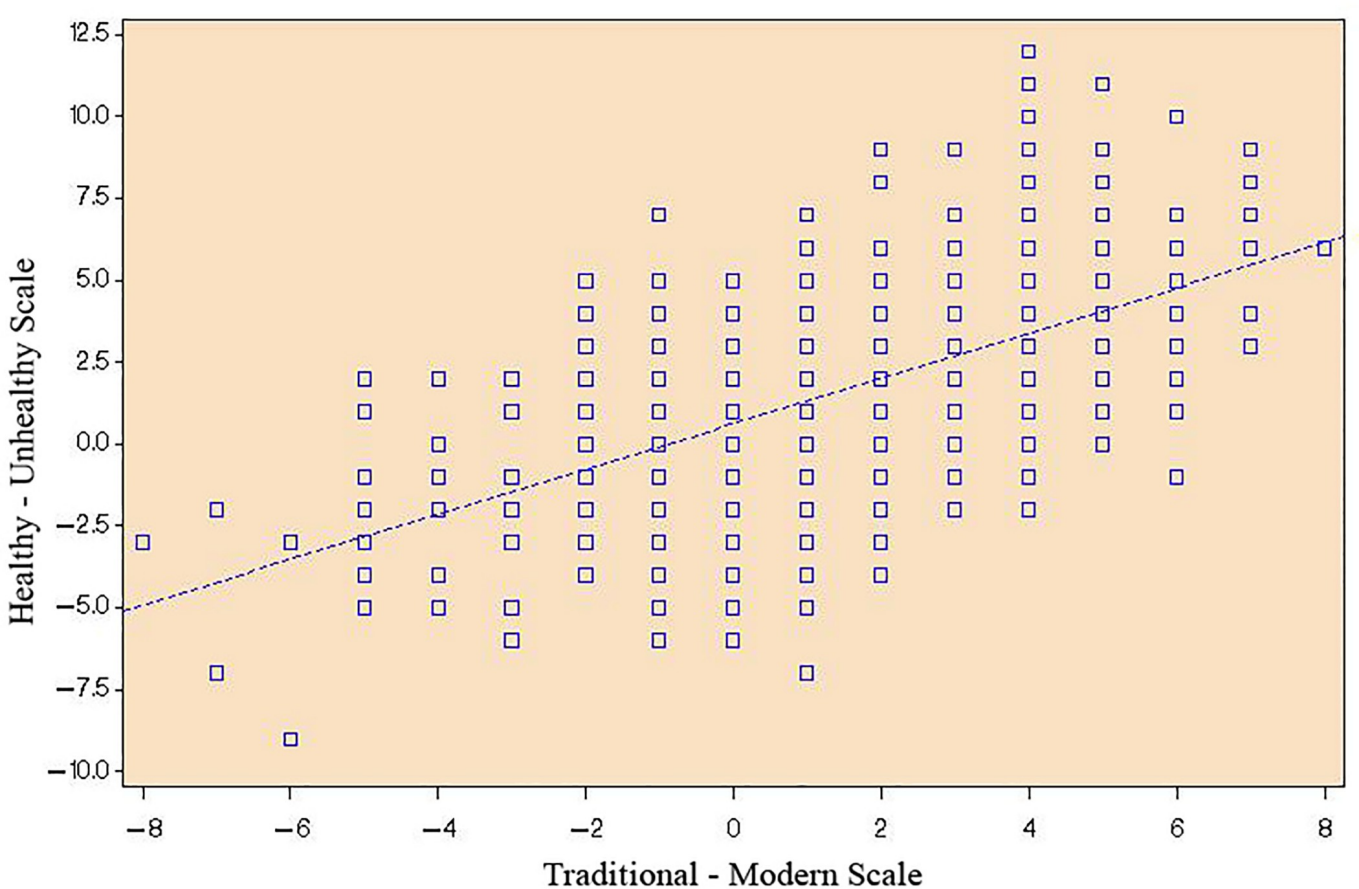
Plot of Traditional-Modern scale scores versus Healthy-Unhealthy scale scores. The plot shows the relationship between the Pohnpeian study participants’ scores on the Traditional-Modern scale and their scores on the Healthy-Unhealthy scale.

The results of our analysis show that the higher the participant scored on the Traditional-Modern Scale (more traditional), the higher the participant scored on the Healthy-Unhealthy Scale (healthier). The relationship appears to be linear (Fig 2). The Healthy/Unhealthy Score = 0.61 + 0.69 (Traditional/Modern Score) (r = 0.56; p<0.0001). The high Traditional Score has a positive correlation with the Healthy Score, while it has a negative correlation with the Modern Score. Similarly the Modern Score has a negative correlation with the Healthy Score and a positive correlation with the Unhealthy Score. There were no statistically significant differences among the municipality categories with respect to age. There is a positive increase of age with higher Healthy Scores and with more Traditional Scores, indicating a tendency for older participants to be living healthier and more traditional lifestyles. The correlation is significant but not as strong as that observed for Healthy/Unhealthy Scores compared to Traditional/Modern Scores.

Remote municipalities have a significantly greater mean Traditional/Modern Score (more traditional) than the main island municipalities, which have a significantly greater score than the transitional municipalities (p<0.0001) (Table 2). Our initial hypothesis was that the transitional municipalities’ scores would lie between the remote and main island municipalities’ scores; however this was not the case. Remote municipalities also have a significantly greater mean Healthy/Unhealthy Score (healthier) than the main island municipalities, which have a significantly greater mean Healthy/Unhealthy Score than the transitional municipalities (p<0.0001) (Table 2). We hypothesized that the transitional municipalities’ score would fall between the remote and main island municipalities’ scores; however this also was not the case. For both scales the transitional municipalities were more similar to the main island municipalities than to the remote municipalities (Table 2).

**Table 2:** Traditional-Modern and Healthy-Unhealthy scale scores of the three Pohnpeian study populations.

Parameter	Main Island (N = 271)	Transitional (N = 57)	Remote Atolls (N = 79)
Age (mean, range)	45.7 (18–90)	47.8 (20–80)	44.4 (20–88)
Percent male/female	45% / 55%	33% / 67%	52% / 48%
Modern/Traditional Score (mean (std))*	0.81 (2.69)	-0.65 (2.25)	2.83 (2.59)
Healthy/Unhealthy Score (mean (std))#	0.99 (3.31)	-0.66 (3.11)	3.73 (2.82)

* The greater the score the more traditional the subject’s lifestyle is considered.

# The greater the score the more healthy the subject’s lifestyle is considered.

In order to further investigate the impact of location, we compared those who lived in the capital of Kolonia to all those living on the main island. Kolonia had a lower Traditional/Modern Score implying a more modern lifestyle and a lower Healthy/Unhealthy Score implying more of an unhealthy lifestyle in the capital, compared to the whole main island sample (Table 3).

**Table 3:** Traditional-Modern and Healthy-unhealthy scale scores of the capital, Kolonia compared with the whole main island sample.

	Main Island			Kolonia (capital)		
Parameter	Trad/Mod	Health/Unhealth	Age	Trad/Mod	Health/Unhealth	Age
N	271			32		
Mean (STD)	0.80 (2.73)	1.00 (3.40)	45.6 (16.40)	-0.38 (2.76)	-0.06 (2.24)	46.5 (13.8)
Median	1.00	1.00	45	0.00	0.00	45
Quartile 1-Quartile 3	-1 : 3	-1 : 3	33–57	-2 : 1	-2 : 2	34–60

Many of our survey questions addressed Pohnpeian diet and food-related practices. We found that 99.5% of our study participants reported eating breadfruit, 97.5% reported eating taro, and 94% reported drinking coconuts, all traditional staples. Frequency and quantity of consumption, however, is unknown. Study participants were also asked if they ate any other green or non-starch vegetables. The five most commonly listed were cucumber (34.5%), eggplant (13.5%), cabbage (13%), carrot (8.5%), and bell pepper (5%). We noted that many others (46%) responded that they eat “any kind of vegetable,” though did not list anything specific. Other individuals (8%) responded that they did not eat any green or non-starch vegetables, and three individuals (<1%) living on the remote Nukuoro atoll said they eat “whatever the ship brings.”

A smaller percentage of transitional Pohnpeians owned land (80%), compared to the main island and remote atoll populations (both 87%) (Table 4). Additionally, a smaller percentage of transitional people were actually growing on their land, growing vegetables, growing fruits, or had their own taro patch (Table 4). Although the Pohnpeians living on the main island were as likely as Pohnpeians living on the remote atolls to own land, a smaller percentage on the main island were actually growing vegetables, fruits, or had their own taro patch compared to those living on the remote atolls (Table 4). Growing food on one’s land requires regular work and physical exertion.

**Table 4:** Agriculture and physical activity in the Pohnpeian study populations.

Parameter	Main Island	Transitional	Remote Atolls
Percent who have land	87%	80%	87%
Percent who grow on their land	88%	78%	97%
Percent growing vegetables	57%	54%	70%
Percent growing fruits	89%	88%	99%
Percent who have a taro patch	87%	75%	99%
Percent who exercise	80%	61%	87%
Percent who go fishing	61%	59%	84%
Percent who harvest coconuts	55%	45%	87%
Percent who have made a canoe	10%	0%	23%
Percent who have helped build a house from traditional materials	30%	11%	67%

We consider this to be one of the measures of exercise, and ultimately health. We found that the highest percentage of Pohnpeians who not only grew on their land, but also exercised, went fishing, harvested coconuts, had made a canoe before, and had helped build a house out of traditional materials, were those living on the remote atolls (Table 4). These activities were less common in the main island population, and even less so in the transitional population (Table 4).

## Discussion

### Transition and health

A large body of research has been conducted on transitional lifestyles and health in the Pacific island of Samoa. Hanna and Fitzgerald [1] administered an extensive health questionnaire to three groups of Samoans: 1) those living in a traditional village in Western Samoa; 2) those living in several villages of modernizing American Samoa; and, 3) those living in urban Honolulu, Hawaii. The most profound differences were found between traditional Western Samoa and the other sites. There were four particular areas of difference, which suggest specific stressors of modernization that may have influences on health. These were changes in the perceptions of food, greater availability of alcohol, wage employment outside of the family, and the increased size of support networks through the inclusion of non-Samoans and non-family members.

Similar to our findings from Pohnpei, the results did not follow the expected pattern of the health of transitional communities being intermediate between village and urban communities. Rather, those living in transitional American Samoa reported higher frequencies of certain symptoms including digestive problems and aches and pains. Another study [2] in Western Samoa, found that negative health outcomes associated with modernization were divided between the intermediate and the most modernized urban communities, when compared to an isolated community that was considered to be the least modernized. Highest serum concentrations of total, high density lipoprotein and calculated low density lipoprotein cholesterol were found in the community considered to be intermediate in level of modernization. However, the highest triglyceride levels were found in the most urbanized and modern community.

Studies in other communities have found that suburban populations are intermediate between those maintaining the traditional lifestyle and those that have moved to urban areas where they live more modernized lifestyles. In a study involving the indigenous Guna, who live on the Panamanian islands, Hollenberg et al. [3] investigated the putative absence of hypertension and age-related rise in blood pressure. The Guna living on the islands showed no age-related rise in systolic, diastolic or arterial pressure. In contrast, the Guna living in Panama City showed a significant rise in blood pressure with age. Suburban Guna were intermediate between the other two groups, but resembled the urban population more than the island population in overall hypertension and age-related rise in blood pressure. Interestingly, the island-dwelling Guna traditionally drink five or more cups a day of flavanol-rich cocoa, shown to improve vascular function in the extremities and kidneys, as well as the blood supply to the brain. The mainland Guna, on the other hand, drink very little cocoa, and what they do drink is commercially available and flavanol-poor [10]. In our study, we also found that within the main island population, those living in the capital of Kolonia, were living less traditional and less healthy lifestyles compared to the whole main island sample (Table 3).

### Diet and health in micronesia

Micronesia’s population is tremendously dependent on other countries for its livelihood and sustenance, particularly concerning imported foods, and has one of the most overweight populations in the world [11]. The World Health Organization survey from 2002 [5] found that 82.7% of the women and 63.9% of the men were classified as overweight (Body Mass Index (BMI)≥25), and 55.8% of women and 30.0% of men were classified as obese (BMI≥30). Over 80% of Pohnpeians surveyed were found to eat less than five combined servings of fruit and vegetables per day; the mean number of fruits per day was 1.6 for women and 1.4 for men, and the mean number of vegetables per day was 2.0 for both genders [5].

Similarly, Cassels [11] found that on Kosrae, another of the four states that comprise the Federated States of Micronesia, 88% of adults over the age of 20 were overweight, 59% were found to be obese and 24% were extremely obese. She suggests that a combination of dietary change, the flow of the global food trade, and dependence on foreign aid stemming from Micronesia’s colonial history of outside rule and outside influence during the past century have all contributed to poor diet and increased obesity rates. These factors and others collectively encourage an excessively sedentary lifestyle, which in itself has major negative health impacts. Cassels explained that food preference may not be enough to ensure healthy diets when imported, often nutrient-poor foods are widely available at low cost. Imported foods may cost less and also require less work than traditional foods harvested from farming or fishing. According to Cassels, an erroneous belief has also developed throughout Micronesia that imported foods are superior to local foods and are thus associated with a higher level of prestige. Purchasing foods requires having money and an income, which are also associated with prestige. Not only are imported food products locally esteemed for their foreign origins, but also for the fact that they need to be purchased.

The activities required for survival in this region naturally included a significant amount of time devoted to agriculture. Raising crops is and has been a highly regarded role and pastime in the culture. As access to pre-prepared foods has increased, there has been less gardening. A local phenomenon indicating wealth is to let tree crops such as breadfruits and mangoes rot on the ground, indicating that the owner can afford “better” food that has been purchased from a local store. Dietary changes have been dramatic with the availability of frozen and canned foods and westernized processed foods. The result has been poorer nutritional status and the prevalence of nutritionally-influenced diseases.

Traditional Pohnpeian diet includes taro (*Colocasia esculenta* (L.) Schott; Araceae), breadfruit (*Artocarpus* spp.; Moraceae), banana (*Musa* spp.; Musaceae), yams (*Dioscorea* spp.; Dioscoreaceae), coconut (*Cocos nucifera* L.; Arecaceae), cassava (*Manihot esculenta* Crantz; Euphorbiaceae), pandanus (*Pandanus* spp.; Pandanaceae) giant swamp taro (*Cyrtosperma merkusii* (Hassk.) Schott; Arecaceae), fish and other seafood, and later dog meat and pork became prized as well [12–14]. Boiled starchy crops made up the traditional staple, while other vegetables were not eaten very commonly. Traditionally fruit and sugarcane were eaten as sweeter snacks (Englberger et al. 2003a).

At present, many highly processed imported foods with elevated levels of fat, salt, and sugar are embedded in the daily Pohnpeian diet, departing radically from the traditional diet. These “new” foods include white rice, white bread, alcohol, candy, doughnuts, soda, canned tuna, spam, corned beef and other canned meats, fatty meats such as frozen chicken parts and turkey tails (that are considered of lesser quality or discards in other regions), heavily processed foods, and frozen vegetables [12,14]. Interestingly, a 58 year old woman, living on the remote atoll of Mwoakilloa who participated in our study shared that she wants Pohnpei to retain its traditional lifestyle because the lifestyle offers “plenty of food to eat” for which the “nutritious value of [the] food is good because all [the] food is fresh,” and asserted that “imported food is giving us disease.” A 45 year old man living in the capital of Kolonia shared that he did not want Pohnpei to continue to become more westernized, “because nowadays we have different kinds of sicknesses,” which he felt were due to lifestyle changes.

Cultural pride can be a considerable motivating factor for high adherence to and acceptability of recommended diet changes, suggesting that the utilization of traditional diet programs can have major implications for improving public health [15]. Traditional food staples including breadfruit, banana, and giant swamp taro have been recommended as culturally appropriate food-based strategies for alleviating malnutrition and vitamin A deficiency [16]. Some Micronesian cultivars of banana, breadfruit, and giant swamp taro contain very high levels of *β*-carotene, the provitamin A carotenoid that contributes most to Vitamin A activity in foods [16]. In bananas, color is a good indicator of provitamin A carotenoid levels, so a simple color guide to estimate carotenoid content could be developed for community selection of cultivars with the most health benefits. Carotenoid levels increase with an increase in the intensity of the banana flesh coloration, from white to yellow to orange [16]. The most striking example is the banana cultivar known as ‘karat’, which can have beta-carotene levels more than a hundred times that of a conventional banana [17]. Vitamin A deficiency in the Micronesian area is among the most prevalent in the world [18] and because of this deficiency, experts believe it created conditions that contributed to an epidemic of the measles in children. Vitamin A supplementation has been suggested for the prevention and treatment of measles-related health complications and fatalities [19].

In other communities, the shift back to a traditional diet, even for a period of a few to several weeks, has been shown to reduce body weight and chronic disease factors. After reversion to a traditional Hawaiian diet for three weeks, Native Hawaiian study participants displayed a decrease in weight, a decrease in systolic and diastolic blood pressures, decrease in serum cholesterol, decrease in low-density lipoprotein, and a reduction of serum triglycerides and glucose levels [15]. The foods selected for the diet were those available in Hawaii before Western contact. Quite similar to the traditional diet on Pohnpei, the Hawaiian study diet consisted of taro, yams, breadfruit, sweet potato, fruit, greens, seaweed, fish, and chicken. In research conducted with Indigenous Australian diabetics [20], reversion to traditional diet for seven weeks showed a significant decrease in fasting glucose, postprandial glucose clearance, plasma insulin concentration and fasting plasma triglycerides. Similar to the study on traditional Hawaiian diet [15], participants reverting to a traditional Australian diet, including hunting and gathering all their food, also displayed significant weight loss. Both studies showed relatively immediate health benefits for study participants who showed positive improvements in health measures after only short-term dietary and lifestyle changes.

It is important to understand that families moving to urban areas in the Western Pacific often face difficulties in continuing to buy and grow all the traditional foods that may have previously been incorporated into their regular diet. One study participant, a 58 year old man on the remote atoll of Mwoakilloa expressed that Pohnpeians are “used to this [traditional] way of life, living off the land,” and felt that “modernization will make life difficult” for them. Furthermore traditional cooking methods such as fat-free cooking in earthen ovens are no longer always possible or practical after relocation to the urban environment, and are typically replaced with cooking methods utilizing fat such as frying and roasting [21].

Access to traditional foods is more than just a health issue in Pohnpei. It is also a food security issue, which makes the case for widely reintroducing traditional foods even stronger. Imported foods may generally seem available, convenient, and affordable; however reliance on such products provides a threat to the food security of Pohnpei. Depending on food produced by other countries creates a system that can be compromised by factors such as storms, rough waters, and changes in currency exchange rates [21].

A 60 year old woman in the capital, Kolonia, shared the opinion of several other study participants when she professed, “We need to depend on ourselves, not depend on others to support us everyday.” Another study participant, a 37 year old man who had moved from the remote island of Mwoakilloa to the capital, Kolonia, feared that if Pohnpei continues to become more westernized, then “Pohnpeians will lose the survival of using the land for farming.” A 59 year old man living on the remote island of Nukuoro explained that traditional Pohnpeian practices are “more natural, this is our lifestyle. Western values disrupt our cycle. We grow our food and make our things without spending money.” A greater percentage of Pohnpeians on the remote atolls grew on their land, grew vegetables and grew fruits than the Pohnpeians who were transitional or living on the main island. In our study, 99% of the participants living on the remote atolls were growing their own patch of taro, a nutritious traditional staple, compared to 87% living on the main island, and only 75% of the transitional individuals (Table 4).

### Traditional lifestyle and knowledge

The link between maintaining health and maintaining knowledge of traditional food varieties, traditional agriculture, and traditional food preparation may seem clear. However, the larger picture involving the connection between maintaining health and maintaining other traditional cultural practices deserves elucidation. Loss of traditional knowledge is a serious and accelerating problem on the global scale. Traditional leaders on Pohnpei have told us that without respect for Pohnpeian culture, there can be no respect for the local environment and thus all the biodiversity it contains.

In an earlier study, our team investigated the loss of traditional practices in a study of canoe-making and other forms of traditional knowledge on Pohnpei [22,23]. The ability to make a traditional canoe depends on both biodiversity and knowledge of local plants—to source the materials used to construct its various parts. Respondents in this study identified at least 27 plant species used in canoe construction. We found that traditional canoe-building knowledge differed significantly among three age classes (under 30, 30–60 and over 60), with knowledge declining from oldest to youngest. Among the youngest age class, only 28% had ever participated in building a canoe. In contrast, 70% of the oldest age class, and 67% of the middle age class had built canoes. Interview responses were used to create individual canoe-knowledge indexes. Several individuals between the ages of 15 and 30 had an index score of zero, whereas all respondents over 30 years of age scored positively on the index.

At current rates of knowledge loss, our study concluded that, without intervention, canoe-making skills could disappear in just one generation. The implications for this significant decline in knowledge go well beyond the loss of an important cultural practice. On Pohnpei, harvesting and managing plants for canoe building aids in maintaining respect for the value of biodiversity. Without this appreciation for the importance of particular plant species used in canoe-making, another local incentive for wilderness conservation disappears along with the canoe-building knowledge [22,23]. Sadly, the scenario of a shift from traditional paddling canoes to imported gasoline powered boats that was described as a threat to maintaining a healthy diet if the price or availability of gasoline increased beyond local people’s ability to pay for it came true over the past few years. According to anecdotal reports this has resulted in less fishing with a consequent reduced harvest of protein sources, with their replacement in the diet by starchy foods. Another issue when using power boats instead of canoes is the problem of engine failure or running out of fuel while at sea. This has led to incidents of mortality tied to fishers going adrift, which is not the case when using a traditional canoe with a sail or paddle. There are many examples of Pohnpeian biodiversity-dependent traditional practices which are eroding rapidly.

The broader implications on health are seen in the connection between knowledge regarding the use of surrounding biodiversity, including not only natural sources of construction materials but also a familiarity with plant-based medicines and wild food varieties. Another major factor in the association of traditional knowledge (and practices) with health involves the physical effort put into many traditional practices. Exercise is an integral part of a traditional lifestyle that involves traditional activities such as farming, fishing, and pounding sakau. Also, less frequent traditional activities such as building a traditional canoe, traditional house, or Nahs (feast house) out of natural materials, require a great deal of physical exertion. Nearly 65% of the Pohnpeian population surveyed by the World Health Organization in 2002 [5] was classified as being physically inactive or with low levels of physical activity; nearly 75% of women and 55% of men.

In our study, a greater percentage of Pohnpeians living on the remote atolls reported exercising, growing food on their land, harvesting coconuts, and going fishing, than those living on the main island or those individuals who were transitional. In fact, the transitional study population had the lowest percentages of individuals engaging in these activities (Table 4). Similarly the population living on the remote atolls had the highest percentage of individuals who had made a canoe or helped to build a house from traditional materials. The main island population had fewer, and the transitional population had the lowest percentage of individuals who had engaged in these physically exerting traditional practices (Table 4).

### Traditional medicine

Our research has identified uses or local names for 44% of all the 1,041 plant species we documented to be found in Pohnpei [7]. Unfortunately, knowledge of these useful plants is decreasing with every passing generation of Pohnpeians. As a region, it is estimated that Micronesia has an extraordinary rate of plant endemism—76% of the native plants are found nowhere else on Earth. Thus many of these plants and the knowledge of their uses do not exist outside of Micronesia. Familiarity with one category of useful plants, the Pohnpeian diversity of traditional plant-based medicines, is integral to maintaining health of the Pohnpeian population. This is especially true for those living on the remote atolls where medical care is particularly limited and travel to the main island is difficult and sometimes impossible.

The importance of retaining traditional medical knowledge and practices has been demonstrated in other areas of the world. In the Bolivian Amazon among the indigenous Tsimané people, maternal ethnobotanical knowledge has been associated with higher indices of child health [24]. Skinfold thickness, height-for-age, and C-reactive protein as an indicator for immunostimulation were measured in 2- to 10-year-old Tsimané children. Each of these child health measures indicated that mothers with higher levels of plant knowledge and usage had healthier children. Individuals with greater levels of ethnobotanical knowledge are likely more efficient in their utilization of local botanical resources including medicinal plants and a diversity of food plants which can better supply the needed micro- and macronutrients. Many rural and remote populations around the world, like the Tsimané, are now facing the challenges as well as opportunities that are connected to globalization. These findings document a potential detriment to human health if traditional plant-based knowledge is lost.

### Medical implications of lifestyle transition on pohnpei and recommendations for healthy living and aging

Of the Pohnpeian populations compared in this study, transitional populations appear to be both living the least traditional lifestyles as well as the least healthy lifestyles. These results may reflect the fact that this population is living under the most pressure impacting their livelihood and general wellbeing. It is likely that transitional populations on the whole do not have as much land for agriculture and building (Table 4) because they have not inherited it by staying in the areas where their other family members reside. They may also be in the poorest economic position and therefore unable to purchase a diversity of healthy foods. In our study, a smaller percentage of transitional people reported exercising than those people living on the main island or the remote atolls (Table 4). Overall the living conditions may not be as good for transitional people since they have not been in their location as long and may not have the community support they had in original communities, including their family and friends.

The importance of community for the health of the individual in Pohnpei is explained by Ward [12]: “In the Ponapean view health is a broad concept. It encompasses the physical and mental aspects of the individual and also the well-being and good fortunes of the groups of which he is a member, especially the family and lineage.” Some Pohnpeians see the connection between westernization and the loss of the traditional sense of community. A 40 year old man living in U told us, “if we are adopting [the] American lifestyle, we will lose our culture. No more supporting others and saying hello to others.” Another 38 year old man living in Kitti explained, “our tradition is good because we can help or support our neighbors or extended families if they need food or water.” A 32 year old man who moved from Sokehs to the remote atoll of Mwoakilloa shared his opinion that, “if we lose our tradition, we will become mean and don’t want to support or help our neighbors.” On the remote atoll of Nukuoro, a 65 year old woman told us that “everyone is together here. We all work together for our wellbeing. One suffers, we all suffer. One has luck, we all have luck.” The growing elderly population of Pohnpei will be in the greatest need of maintaining community networks and social relationships as younger family members continue to migrate to urban areas or outside of Pohnpei entirely.

Based on the results of our survey, and our knowledge of traditional life on Pohnpei, we suggest the following several health and lifestyle recommendations be considered for adoption by all islanders or if already in place, intensified to the degree possible: (1) Maintain and encourage traditional group cultural activities, especially those which are multi-generational, to strengthen community, family, and clan relationships; (2) Maintain and encourage traditional practices utilizing knowledge pertaining to local biodiversity. This will promote the conservation of endemic and native biodiversity, and the foods, medicine, and natural materials that the Pohnpeian environment provides; (3) Maintain and encourage traditional practices requiring physical exertion, such as farming, fishing, canoe building, and traditional construction, particularly those that involve outdoor activity. This will keep body weights down and help decrease incidences of chronic diseases; (4) Document, maintain and revitalize practices of traditional medicine and provide educational opportunities for the population at large to learn about traditional home remedies and medicinal plants. This can provide sustainable healthcare in remote areas as well as be a “first line of defense” in the home against minor illnesses and injuries throughout Pohnpei; (5) Utilize traditional sakau preparation and consumption methods to the extent possible. This may encourage traditional use rather than encourage sakau as a recreational activity; (6) Return to traditional diet and traditional cooking methods whenever possible. This will maximize sustainable food sourcing, physical activity and cultural respect for traditional dietary practices; (7) Consume more low fat animal foods, more fresh seafood and less canned meats. This will provide high quality protein containing less sodium; (8) Grow a diverse array of vegetable and fruit varieties in home and community gardens. This will maximize access to a robust nutrient base.

Findings from our study on the island of Pohnpei have important implications that are applicable to the greater global population, including the United States. Chronic diseases, including heart disease, cancer, stroke and diabetes account for 7 out of 10 deaths among Americans each year, and are among the most preventable of all health problems [25]. The health issues found among those aging in the developing world are largely mirrored in the older populations of the world’s richest nations. Prevalence of chronic diseases such as stroke and hypertension among the older population in urban areas of Santo Domingo, capital of the Dominican Republic, a small island country in the Caribbean, was found to be similar to that among the population of the same age in the United States [26]. These critical health concerns are shared around the world, in the developing and developed world alike.

There has been an explosion in the size of the population of Americans over 65 years of age since 1900, which grew from 3.1 million to 35 million in 2000 [27]. In 2011 the U.S. population shifted to an older age profile as the first Baby Boomers turned 65. By 2030 the older population is projected to be twice as large as it was in 2000, representing nearly 20 percent of the total American population [27]. The prevalence of chronic age-related diseases will likely increase with the demographic aging of the American population. Among older Americans, individuals may be vulnerable to food insecurity, the limited or uncertain access to nutritious foods, and paradoxically the obesity associated with it. This is mediated in part by the physical limitations caused by weight-related disabilities, which may limit people’s ability to travel and shop for, and prepare nutritious food [28]. Isolation, living alone, limited social networks, geographical limitations to accessing nutritious food, and low income may be other contributing factors to food insecurity, and therefore obesity and other chronic diseases among older people in the U.S.

Divorce, widowhood, job loss, retirement, and relocation are transitions associated with aging, as well as with times of economic hardship. Half of the marriages in the U.S. end in divorce [29]. Divorced and widowed American women were shown to increase their physical activity, however they also had more than a twofold increased risk of starting smoking or starting smoking again, and had a decrease in vegetable intake relative to women who stayed married [30]. Divorced and widowed American male health professionals also were shown to have a decrease in vegetable intake, and widowed men had an increase in alcohol consumption [31]. A study on 55–69 year old men exiting the labor force in the 1980’s found to this work transition be associated with poor health, especially so for younger men, and men who had working wives [32]. Populations in transition may be among the most vulnerable, including those moving to the U.S., and those moving to urban areas within the U.S. The U.S. is a nation of people in transition, a nation of immigrants and emigrants, of young and old faced with frequent life and career changes.

Though the traditional lifestyles of those living on remote Pohnpeian atolls may not be applicable in every sense to the North American population in its entirety, there are lessons to be learned regarding the health benefits of the diverse nutritious foods, regular physical exercise, and community relationships so integral to the Pohnpeian traditional way of life that are widely applicable to the global population “beyond the reef” as the Pohnpeians refer to the rest of the world. We must also be aware of the challenges posed to human health and quality of life of a people in transition, whose roots may not be as deep as their forbearers. Lessons from this remote island can be useful in understanding healthy living, aging and longevity elsewhere in the world. Importantly, the process of transition must be recognized as a significant lifestyle and health risk and given the same attention that we give to other risk factors that negatively influence our health and quality of life.

## Supporting information

S1 File Permission to participate forms in English and Pohnpeian. (PDF)

S2 File Survey questions in English and Pohnpeian. (PDF)
