# Supplementary material for: Traditional lifestyles, transition, and implications for healthy aging: An Example from the remote island of Pohnpei, Micronesia
Source: PLoS One. 2019 Mar 12;14(3):e0213567. doi: 10.1371/journal.pone.0213567 (PMC6413935; doi:10.1371/journal.pone.0213567)
Supplement: S1 File — (PDF) [file pone.0213567.s001.PDF]

Permission to Participate Form

Pohnpei Lifestyle and Healthy Aging Survey

We are conducting a survey of people on Pohnpei with the goal of learning how various life style factors such as food, community and family relationships, exercise and other daily physical activity, and sleep contribute to health as a person ages. Participation in this survey is completely voluntary on your part. We request that you answer some questions for this survey, by filling out the form which you will be provided. Please ask the person who gave you this form if you have any questions. All information will be kept confidential as to the specific identity of the participants. The information collected in this survey will be used to help develop healthy lifestyle recommendations for the Pacific region as well as other parts of the world.

We would greatly appreciate and value your contribution to this survey. By granting us permission to include your information in this survey, we also wish to be able, as appropriate, to examine your medical records in the local hospitals and clinics, along with other relevant data that might help us identify diseases and other medical conditions related to aging, which can be influenced by living a healthy lifestyle as outlined above.

This survey is part of the Ethnobotany of Pohnpei Project that has as its goal the study of local traditional culture as it relates to the uses of plants for many purposes, including food and medicine, with the goal of conservation of this knowledge, along with the biodiversity of the natural environment that it is based upon. This survey is sponsored by The New York Botanical Garden.

By signing below you grant us permission to include you in the survey.

Thank you very much.

THE POHNPEI ETHNOBOTANY PROJECT

Agreed and Accepted,

Name:

Date:

Project en Tuhke oh aramas nan Micronesia
Kisin likou en mweimwei en patehng doadoahk wet

Se patohwen wiewia roprop ehu me pid tuhke en Micronesia, oh soahng kan me tuhke kin kak doadoahk ohng pwehn kak wia tetehn doadoahk ohng wiawidahn pwuhk ehu me oaralepe pahn *Project en tuhke kan oh aramas en Micronesia* pwehn sewese koaros en kak wehwehkihla tuhke kan me dierek nan Micronesia, kaoros me wia pwilidak oh me patohlongodo kan, oh kateparail me kedeudeudo kan oh doadoahkparail kan ohng nan mwehi wet. Se patowen weliwelian pali kapatapat kei nan government oh me pil sohte pato pahn government me pato Pohnpei oh likin Pohnpei, oh songosong doadoahkpene pwe sen kak en patohwanpene oh kolokol soahng kesempwal kan me kitail ketin mwahngih me pid kesempwal en tuhke pwukat opil songsongen kepikipik kan me wia poason pen wehwe wet, sang ni aht utung duwen doadoak keneinei me konehng kitail en wiaiong kepikipik kesempal kei me mie rehtail. Kosonned pil anahne pohnese oh wauhniki ire pwukat pwe en wia poason en tetehn kasukuhl kan. Pali kan me katangatanga project wet nan Micronesia iei me iangahki Conservation Society of Pohnpei CSP, The New York Botanical Garden, palih kei nan Kapworment en Pohnpei, Lapalap oh semen kei nan Tiahk, College en Micronesia, Continuum Center for Health and Healing me pato Beth Israel Hospital nan wein New York City, Program for Integrative Medicine me pato University en Arizona, The nature Conservancy, The National Tropical Botanical Garden, Belau National Museum oh pil iangahki Belau Office of the Council of Chiefs.

Sang mweimwei me alahldier sang nan pali en government oh tiak, se pahn pil raparapahki soangen tuhke kei sang wasah kei nan Micronesia. Tuhke me pahn dierekdasang nan roprop wet pahn idaidpene, peleng oh pekederlahng semen kan pwe ren kadehede soangen tuhke pwukat, oh pahn pil wiala kisehn iengen tetehn kasukuhl en tuhke, me pato College of Micronesia, New York Botanical Garden oh pil iangahki National tropical Botanical Garden. Soagen tuhke oh pil mengihtikpe karoas me dierekdahsang ni project wet pahn dodoahk ong mehn kasukuhl ahpw kaiden mehn wie mwoni de netla oh pahn pil wia mehn sawas de kaweid nan Micronesia oh pil wasahkan me pil men iang ese duwen tuhke pwukat ni e pahn sohte isaihs. Mengihtik karoas me alahldi ohng wiepen dodoanki tuhke pwukat pahn intingdi oh kileledi nan pwuhk ehu me pahn wiawihda. Se pil raparapahki ire oaralap ohng ni dodoangki tuhke ong mehn karoson, wini, wie ihmw, soai, likou oh pil mehn ieias teikan. Se sohte kolokolpene mehkot me rir ni aht wiewia mwomwen dodoak wet ahpw se sohte kasik kumwail en kasalehda mehkot me wia mehkot kesempwal ong ehu kouson. Ni eh sansalehr powe, kawehwehpen tuhke oh tuhke kan me pahn alahldi nan roprop wet pahn iang pato nan pwuhk me pida ire wet nan momour en Micronesia, ni eh kileledier de intingdier ong dih en met opil dih mwuhr.

Ma komwi iang sapwelimanki aht tungoal kapwunod me pid momour de tiahk en Micronesia oh doadoahk kedeudeu kan en tuhke kan a se patohwen kasamwoh sapwelimomwi tow nan project wet. Mwaromwi pahn kileledi iangahki tuhke kan me kitail ketin sawaspenehr oh diarada pahn patehng kaweid de mengihtik kan me komwi sawaski oh iang pato nan pwuhk me pahn wiawihda.

Ma komwi sain pah, komwi mweidadahr sen kapeidak komwi nan project wet.

Kalahngan en kupwuromwi.

Mweidada oh ketiki,

Mwaromwi:
Rahn: F [illegible]
