# Supplementary material for: Traditional lifestyles, transition, and implications for healthy aging: An Example from the remote island of Pohnpei, Micronesia
Source: PLoS One. 2019 Mar 12;14(3):e0213567. doi: 10.1371/journal.pone.0213567 (PMC6413935; doi:10.1371/journal.pone.0213567)
Supplement: S2 File — (PDF) [file pone.0213567.s002.pdf]

DEMOGRAPHIC QUESTIONS

1. What is your name?<br>Title?	
2. Male ☐ Female ☐	
3. Which municipality are you from?	
4. How old are you?	
5. Were you born in a hospital? Yes ☐ No ☐	
6. How much education have you completed? Grade school? ☐<br>Middle School? ☐ High School? ☐ College? ☐	
7. Are you single ☐ married ☐ divorced/separated ☐ widow(er) ☐	
8. If married, do you have children Yes ☐ No ☐	How many? ☐
9. Are your parents or your in-laws living with you? Yes ☐ No ☐	
10. Are there married children living with you? Yes ☐ No ☐	
11. How many brothers and sisters do you have? Male ☐ Female ☐	
12. Have you ever traveled off the island?<br>Yes ☐ No ☐<br>If yes, for how long?	13. Where did you travel to?
14. Lived off the island<br>Yes ☐ No ☐ If yes, where and for how long?	15. For work or school?<br>Yes ☐ No ☐ If yes, where and for how long?
16. What is your Nahnmwarki's name?	
17. Who is the president of the Federated States of Micronesia?	
18. Do you own a car/truck? Yes ☐ No ☐	

19. If no, is this important to have? Yes ☐ No ☐	
20. Are you working on getting a car? Yes ☐ No ☐	
21. Do you share a car/truck? Yes ☐ No ☐	
22. Do you own a boat? Yes ☐ No ☐	23. Do you share the use of a boat? Yes ☐ No ☐
24. How often do you use the boat? Daily ☐ Weekly ☐ Monthly ☐	
25. Do you have a traditional canoe? Yes ☐ No ☐	
26. If yes, did you make it? Yes ☐ No ☐	
27. How old is it? ☐	
28. Does someone in your family have a canoe? Yes ☐ No ☐	
29. Does someone (you or a family member) know how to make a canoe? Yes ☐ No ☐	
30. Who is it? How related?	
31. Do you fish? Yes ☐ No ☐	32. How often do you fish? Every day ☐ Several times a week ☐ Once a month ☐
33. Do you sell fish Yes ☐ No ☐	
34. Are there any rules from the Nahnmwarki that you must follow for fishing? Yes ☐ No ☐	

35. Do you eat canned fish? Yes ☐ No ☐	36. How many cans a week?	
37. Do you eat fresh fish? Yes ☐ No ☐	38. How often?	
39. Do you raise pigs? Yes ☐ No ☐	40. For selling? Yes ☐ No ☐	41. For eating? Yes☐ No ☐
42. Do you raise chickens? Yes ☐ No ☐	43. For selling? Yes ☐ No ☐	44. For eating? Yes☐ No ☐
45. Do you have a piece of land? Yes ☐ No ☐	46. Do you live on it? Yes ☐ No ☐	
47. Do you have any land in the mountain forest? Yes ☐ No ☐		
48. In the lowland? Yes ☐ No ☐		
49. Are you growing anything on your land? Yes ☐ No ☐		

50.	What are you growing? Kava? Yes ☐ No ☐<br>Vegetables? Yes ☐ No ☐
51.	Fruits? Yes ☐ No ☐ Trees? Yes ☐ No ☐
52.	What is your house made of: wood? Yes ☐ No ☐<br>Traditional materials? Yes ☐ No ☐<br>Cement? Yes ☐ No ☐
53.	Do you work? Yes ☐ No ☐
54.	What do you do?
55.	Are you working for the government? Yes ☐ No ☐
56.	Do you watch television? Yes ☐ No ☐<br>If yes, how many hours per week ☐
57.	Do you own a television? Yes ☐ No ☐
58.	Do you wish Pohnpei was more like the United States or like Hawaii?<br>Yes ☐ No ☐
59.	If so, why?
60.	If not, why?
61.	Do you wish Pohnpei could retain most of its traditional lifestyle and values?<br>Yes ☐ No ☐
62.	If so, why?

63. If not so, Why not?
64. Do you drink sakau? Yes ☐ No ☐
65. Do you drink sakau on the rock? Yes ☐ No ☐ If yes, how many cups?
66. Do you pound your own sakau? Yes ☐ No ☐
67. Do you drink every day? Yes ☐ No ☐ If not, how many times per week?
68. Do you go to sakau bars? Yes ☐ No ☐
69. How many bottles do you drink in a sitting? 1 ☐ 2-5 ☐ 5+ ☐
70. Do you drink alcohol with the sakau? Yes ☐ No ☐
71. All the time? Yes ☐ No ☐
72. Do you buy sakau? Yes ☐ No ☐
73. How often do you drink?
74. Have you ever taken sakau pills? Yes ☐ No ☐
75. Are they stronger ☐ or weaker? ☐
76. Did you ever get the sakau skin? Yes ☐ No ☐
77. Do you eat taro? Yes ☐ No ☐
78. Do you have a taro patch? Yes ☐ No ☐
79. Do you eat breadfruit? Yes ☐ No ☐
80. Do you eat white rice? Yes ☐ No ☐
81. Do you eat pizza? Yes ☐ No ☐
82. Do you eat candy? Yes ☐ No ☐
83. What kinds of (green/non-starch) vegetables do you eat?
84. From the Garden? Yes ☐ No ☐
85. From the can? Yes ☐ No ☐
86. Fresh vegetables from the store? Yes ☐ No ☐
87. Do you eat bread? Yes ☐ No ☐
88. White bread? Yes ☐ No ☐
89. Whole wheat bread? Yes ☐ No ☐
90. Do you eat spam? Yes ☐ No ☐
91. How many cans a week?
92. Do you consider yourself healthy? Yes ☐ No ☐
93. When was the last time you saw your doctor?

94.	Was it for a regular checkup? Yes ☐ No ☐<br>or for a medical problem? Yes ☐ No ☐
95.	Do you go to a private clinic for care? Yes ☐ No ☐<br>To the hospital Yes ☐ No ☐ Or to a dispensary? Yes ☐ No ☐
96.	Do you use traditional/local medicine? Yes ☐ No ☐
97.	Do you use local medicine for: Cough ☐ Fever ☐<br>Men's/Women's sickness ☐ Arthritis ☐<br>____________________<br>____________________
98.	What kind of sickness do you have?

99.	Do you chew betelnut? Yes ☐ No ☐
100.	How often do you chew?
101.	Do you chew with lime? Yes ☐ No ☐
102.	Do you chew with leaf? Yes ☐ No ☐
103.	For how many years have you chewed?
104.	Do you drink coconuts? Yes ☐ No ☐
105.	Do you harvest your own coconuts? Yes ☐ No ☐
106.	Do you work on a computer? Yes ☐ No ☐
107.	Do you type? Yes ☐ No ☐
108.	Do you have friends? Yes ☐ No ☐
109.	How often do you talk to them?
110.	Do you see them at the sakau bar? Yes ☐ No ☐
111.	Do you work at a desk job? Yes ☐ No ☐<br>Awa depe?
112.	Do you rent videos? Yes ☐ No ☐
113.	Every day? Yes ☐ No ☐
114.	How many hours?
115.	How many hours do you sleep per night?
116.	How many hours do you sleep on sakau?
117.	How many hours do you sleep without sakau?
118.	Do you sleep better with sakau?
119.	Do you exercise
120.	If yes, what do you do?
121.	Do you help build houses or nahs?

SURVEY EN ROSON MWAHU

1. Ia mwaromwi souleng?
2. Ohl de Lih?
3. Mehnia wehi me komw ketsang ie?
4. Komw sounpar depe?
5. Komw ipwidi nan imwen wini? Ehi ☐ Soh ☐
6. Mehnia school me komw neksang ia? Komw lel pwihn kedepe? ☐
High School? ☐ College? ☐
7. Keke … Kiripw ☐ Pwopwoud ☐ Mweipeseng/Tohrohrpeseng ☐ Liohdi ☐
8. Mie sapwelimomwi seri? Ehi ☐ Soh ☐ — Meh depe? ☐
9. Omw pahpa nohno de en omw pwoud ah pahpa nohno kin koukousoan rehmwi?
Ehi ☐ Soh ☐
10. Mie sapwelimomwi seri me pwopwoud ahpw koukousoante rehmwi? Ehi ☐
Soh ☐
11. Meh depe riomwi pwutak de/oh serepein? Ohl depe ☐ Lih depe ☐
12. Mie pak komw seilok la liki? Ehi ☐ Soh ☐
13. Ma ehi, ia wen werei? ☐
14. Mie wasa komw kouson ie likin Pohnpei? Ehi ☐ Soh ☐ Ma ehi, ia wasa oh ia wen werei?
15. Ia wasa komw seilok la ie?
16. Ia lengileng en omwi Nahnmwarki?
17. Ia eden President en Wehin FSM?
18. Mie tehn weremwi sidohsa? Ehi ☐ Soh ☐
19. Ma soh, e kesempwal komwi en tehn werenki sidohsa? Ehi ☐ Soh ☐
20. Komw kin nannantih en ale sidohsa ansou wet? Ehi ☐ Soh ☐
21. Komw kin iang doadoahnki tehn weren emen aramas sidohsa? Ehi ☐ Soh ☐
22. Mie tehn weremwi pwoht? Ehi ☐ Soh ☐
23. Komw kin doadoahki tehn weren emen aramas pwoht? Ehi ☐ Soh ☐
24. Pak depe komw kin doadoahki pwoht? Rahn koaros ☐
Pak ehu nan wihk ehu ☐ Pak ehu nan sounpong ehu ☐
25. Mie weremw wahr? Ehi ☐ Soh ☐

26. Ma ehi, komwi me wiahda? Ehi ☐ Soh ☐		
27. Ia wen werein tehn weremwi wahren?		
28. Mie tohn omwi peneinei kin werenki wahr? Ehi ☐ Soh ☐		
29. Mie tohn omwi peneinei kin ese wiahda wahr? Ehi ☐ Soh ☐		
30. Ihs?		
31. Komw kin laid? Ehi ☐ Soh ☐	32. Pak depe komw kin laid? Rahn koaros ☐<br>Ekeipak nan week ehu ☐<br>Pak ehu nan ehu sounpwung ☐	
33. Komw kin netki mwahmw? Ehi ☐ Soh ☐		
34. Mie tiahk en wiepen laid me komw kin mwangih oh idawen? Ehi ☐ Soh ☐		
35. Komw kin koanoate de sakan poahten mwahmw? Ehi ☐ Soh ☐	36. Pwoaht depe nan ehu week?	
37. Komw kin koanoate de sakan mwahmw me laidida? Ehi ☐ Soh ☐	38. Pak depe nan ehu wihk?	
39. Komw kin apwaliada pwihk? Ehi ☐ Soh ☐	40. Mehn netila? Ehi ☐ Soh ☐	41. Mehn mwenge? Ehi☐ Soh ☐
42. Komw kin kakairada malek? Ehi ☐ Soh ☐	43. Mehn netila? Ehi ☐ Soh ☐	44. Mehn mwenge? Ehi☐ Soh ☐
45. Mie nin limwomwi sahpw? Ehi ☐ Soh☐	46. Komw kin kousousoan loale? Ehi ☐ Soh ☐	
47. Mie nin limwomwi sahpw nanwel? Ehi ☐ Soh ☐		
48. Mie ninlimwomwi sahpw nansapw? Ehi ☐ Soh ☐		
49. Mie wahnsapw komw kin poadok nan ninlimomwi sahpw? Ehi ☐ Soh ☐		
50. Dahme komw kin poadok? Sakau? Ehi ☐ Soh ☐ Iasai? Ehi ☐ Soh ☐		
51. Wahn tuhke? Ehi ☐ Soh ☐ Tuhke? Ehi ☐ Soh ☐		
52. Dahme tehnpahsemwi wiawihkida: Tuhke? Ehi ☐ Soh ☐<br>Dipwisou en Pohnpei? Ehi ☐ Soh ☐<br>Cement (siment)? Ehi ☐ Soh ☐		
53. Komw kin doadoahk? Ehi ☐ Soh ☐		

54. Songen doadoahk da me komw kin wia?

55. Komw kin doadoahk ong government? Ehi ☐ Soh ☐

56. Komw kin mahsani TV? Ehi ☐ Soh ☐
Ma ehi, awa depe nan ehu week? ☐

57. Mie sapwelimwomi TV? Ehi ☐ Soh ☐

58. Komw kupwuriki Pohnpei en duehte United States de Hawaii? Ehi ☐ Soh ☐

59. Ma ehi, pwehda?

60. Ma soh, pwehda?

61. Komw kupwuriki Pohnpei en kolokolete tiahk oh soangen wiepen ieias me kedekedeudeu doh?
Ehi ☐ Soh ☐

62. Ma ehi, pwehda?

63. Ma soh, pwehda?

64. Komw kin koanoate de sakan sakau? Ehi ☐ Soh ☐

65. Komw kin iang konot sakau pohn takai? Ehi ☐ Soh ☐ Mah ehi, ah ngarangar depe komw kin sakanla de nimala?
66. Komw kin sukidi pein koanoat omwi de sekemwi sakau? Ehi ☐ Soh ☐
67. Komw kin sakau rahn koaros? Ehi ☐ Soh ☐<br>Ma soh, ah ak depe nan week ehu?
68. Komw kin ketla iang marked en Sakau kan? Ehi ☐ Soh ☐
69. Kep depe komw kin koanoate de sakanla? 1 ☐ 2-5 ☐ 5+ ☐
70. Komw kin koanoate de sakan sakau en wai kapatahieng sakau en Pohnpei?<br>Ehi ☐ Soh ☐
71. Ansou koaros? Ehi ☐ Soh ☐
72. Komw kin pwainda sakau? Ehi ☐ Soh ☐
73. Nan week ehu pak depe komw kin iang sakau?
74. Komw iangehr sohng soangen sakau me pato ni weren wini ko? Ehi ☐ Soh ☐
75. E kehlail sang sakau en Pohnpei? ☐ de luetesang? ☐
76. Mie pak me nin limwomwi kihl kin ohkihla sakau? Ehi ☐ Soh ☐
77. Komw kin koanaote de sakan mwahng? Ehi ☐ Soh ☐
78. Mie sapwelimwomwi lehpwel? Ehi ☐ Soh ☐
79. Komw kin koanaote de sakan mahi? Ehi ☐ Soh ☐
80. Komw kin koanaote de sakan rice? Ehi ☐ Soh ☐
81. Komw kin koanaote de sakan pizza? Ehi ☐ Soh ☐
82. Komw kin koanaote de sakan candy? Ehi ☐ Soh ☐
83. Songen iasai da me komw kin koanaote de sakan ?
84. Sang ni omwi mwetiwel? Ehi ☐ Soh ☐
85. Sang nan pwoht? Ehi ☐ Soh ☐
86. Sang nan freezer? Ehi ☐ Soh ☐
87. Ke kin koanaote de sakan pilawa? Ehi ☐ Soh ☐
88. Pilawa pwetepwet? Ehi ☐ Soh ☐
89. Pilawa whole wheat? Ehi ☐ Soh ☐
90. Komw kin koanaote de sakan Spam? Ehi ☐ Soh ☐
91. Pwoht depe nan ehu week?
92. Komw pepehm me komw rohson? Ehi ☐ Soh ☐
93. Iahd imweseklahn omwi tuheng toakte (doctor)?
94. Komw tuhkiheng toahkte pwehki komw men pein tehkada roson en palinweremwen? Ehi ☐ Soh ☐<br>De komw udahn luhmwuhmw oh anahne tuheng toahkte? Ehi ☐ Soh ☐
95. Komw kin kohla ni private clinic? Ehi ☐ Soh ☐ de ni imwen wini laud<br>Ehi ☐ Soh ☐ de ni dispensary? Ehi ☐ Soh ☐

96. Komw kin doadoahki wini en Pohnpei? Ehi ☐ Soh ☐
97. Soangen wini da me komw kin doadoahki?
98. Ma ehi, ohng luhmwumw da?
99. Komw koanoate de sakan pwuh? Ehi ☐ Soh ☐
100. Nan rahn ehu pak depe komw kin koanaote de sakan pwuh?
101. Komw kin koanoate de sakan pwuh oh pweht patpene? Ehi ☐ Soh ☐
102. Komw kin koanoate de sakan kapwoi? Ehi ☐ Soh ☐
103. Pahr depeier komw kin koanokoanoate de sakasakan pwuh?
104. Komw kin koanoate de sakan uhpw? Ehi ☐ Soh ☐
105. Komw kin pein dauridi koanoat omwi de sekemwi uhpw? Ehi ☐ Soh ☐
106. Komw kin doadoahki computer? Ehi ☐ Soh ☐
107. Komw kin type? Ehi ☐ Soh ☐
108. Mie koampoakepahmw? Ehi ☐ Soh ☐
109. Iawen dod en kumwail kin tupene oh mahmahsen?
110. Komw kin tuheng irail ni wasahn market en sakau en Pohnpei kan? Ehi ☐ Soh ☐
111. Komw kin doadoahk nan ohpis? Ehi ☐ Soh ☐
Awa depe?
112. Komw kin iang rent video kaset? Ehi ☐ Soh ☐
113. Rahn koaros? Ehi ☐ Soh ☐
114. Awa depe?
115. Komw kin mehir awa depe ni pwong ehu?
116. Ma komw kin sakau, komw kin mehir awa depe?
117. Ma komw sohte sakau, komw kin mehir awa depe?
118. Omwi meir kin mwahusang ansou komw kin iang sakau sang ni omw sohte iang sakau?
119. Komw kin exercise?
120. Ma ehi, soangen exercise da?
121. Komw kin iang wiahda ihmw de nahs?
